# Monocyte adhesion to and transmigration through endothelium following cardiopulmonary bypass shearing is mediated by IL-8 signaling

**DOI:** 10.3389/fcvm.2024.1454302

**Published:** 2024-12-11

**Authors:** Hao Zhou, Marta Scatena, Lan N. Tu, Cecilia M. Giachelli, Vishal Nigam

**Affiliations:** ^1^ Department of Bioengineering, University of Washington, Seattle, WA, United States; ^2^ Seattle Children’s Hospital, Seattle, WA, United States; ^3^ Department of Pediatrics, University of Washington, Seattle, WA, United States

**Keywords:** cardiopulmonary bypass, monocytes, endothelial cells, IL-8, shear stress

## Abstract

**Introduction:**

The use of cardiopulmonary bypass (CPB) can induce sterile systemic inflammation that contributes to morbidity and mortality, especially in children. Patients have been found to have increased expression of cytokines and transmigration of leukocytes during and after CPB. Previous work has demonstrated that the supraphysiologic shear stresses existing during CPB are sufficient to induce proinflammatory behavior in non-adherent monocytes. The interactions between shear stimulated monocytes and vascular endothelial cells have not been well studied and have important translational implications. With these studies, we tested the hypothesis that non-physiological shear stress experienced by monocytes during CPB affects the integrity and function of the endothelial monolayer.

**Methods:**

We have used an *in vitro* CPB model to study the interaction between THP-1 monocyte-like cells and human neonatal dermal microvascular endothelial cells (HNDMVECs). THP-1 cells were sheared in polyvinyl chloride (PVC) tubing at 2.1 Pa, twice of the physiological shear stress, for 2 h. ELISA, adhesion and transmigration assays, qPCR, and RNA silencing were used to assess the interactions between THP-1 cells and HNDMVECs were characterized after co-culture.

**Results:**

We found that sheared THP-1 cells adhered to and transmigrated through the HNDMVEC monolayer more readily than static THP-1 controls. Sheared THP-1 cells disrupted the VE-cadherin and led to the reorganization of cytoskeletal F-actin of HNDMVECs. A higher level of IL-8 was detected in the sheared THP-1 and HNDMVEC co-culture medium compared to the static THP-1 and HNDMVEC medium. Further, treating HNDMVECs with IL-8 resulted in increased adherence of non-sheared THP-1 cells, and upregulation in HNDMVECs of vascular cell adhesion molecule 1 (VCAM-1) and intercellular adhesion molecule 1 (ICAM-1). Finally, inhibition of HNDMVECs CXCR2/IL-8 receptor with Reparixin and of IL-8 expression with siRNA blocked sheared THP-1 cell adhesion to the endothelial monolayer.

**Conclusions:**

These results suggest that CPB-like sheared monocytes promote IL-8 production followed by increased endothelium permeability, and monocyte adhesion and transmigration. This study revealed a novel mechanism of post-CPB inflammation and will contribute to the development of targeted therapeutics to prevent and repair the damage to neonatal patients.

## 1 Introduction

Cardiopulmonary bypass (CPB) is a technique that maintains blood circulation in an extracorporeal circuit, in which blood is propelled by mechanical pumps and flows in plastic tubing. This technique provides the surgeon with a bloodless field to conduct open-heart surgery, while minimizing the ischemic damage to the organs. However, since the blood components are exposed to an environment that is very different from the native blood vessels during CPB, the incidence of complication occurs at a rate of 29.2% (1). These complications can lead to systemic inflammation and multiorgan dysfunction, which translates to a mortality rate of 10.7% in pediatric patients. Although modifications have been made to suppress the inflammatory response through corticosteroid administration, coating the CPB tubing, and filtering the blood postoperatively, no significant improvement has been reported to date (2–6). At present, finding a CPB-specific pathway that leads to the inflammatory response in pediatric patients is the key to reducing the complications and mortality rate.

Post-CPB complications are largely associated with the secretion of inflammatory cytokines, such as IL-1β, IL-6, IL-8, and Tumor necrosis factor alpha (TNF-α) during and after bypass (7–16) and disruption of the endothelial barrier function (17, 18). However, most current studies are based on clinical samples, which are unlikely to decouple the effects of the CPB and the surgical intervention. Therefore, it remains challenging to find targeted pathways that prevent the inflammatory response specific to CPB. Given that blood cells can experience three times more shear stress in a CPB circuit compared to that in the native blood vessel (19), studying the correlation between CPB shear and cytokine released by cells can help unveil CPB-specific pathways. Recently, we developed an *in vitro* model that was designed to distinguish the non-CPB factors such as surgical trauma, blood transfusion, drug administration, and the specific condition of the patient from CPB-specific inflammatory changes (19). Using this model, we demonstrated that CPB shear stress specifically upregulated the expression of IL-8 and TNF-α in monocytes, as well as giving rise to a subpopulation of monocytes undergoing TNF-α-mediated necroptosis. While the TNF-α-mediated inflammatory response of cells in the CPB setting has been well-studied (19–21), the role of IL-8 in regulating the cellular response after CPB is still unclear.

IL-8 is a chemoattractant that regulates the recruitment of neutrophils, basophils, and T-cells during inflammation. Cells that can secrete IL-8 include monocytes, macrophages, neutrophils, and endothelial cells. The receptors for IL-8, CXC chemokine receptor (CXCR)1 and CXCR2, are also reported to be present in a wide range of cell types, including endothelial cells, monocytes, neutrophils, and fibroblasts, with the former having more specific affinity to the IL-8 than the latter (22). Evidence has shown that in CPB patients, perioperative plasma IL-8 level is positively correlated to the length of inotropic support, length of mechanical ventilation, and incidence of acute kidney injuries (23–27). Thus, IL-8-mediated signaling pathways may play an important role in the onset of CPB-induced inflammatory response at the cellular level. In this study, we used a novel *in vitro* CPB model to characterize the molecular mechanism of how leukocytes affected by CPB shear can induce inflammatory response in endothelial cells. We identified the IL-8/CXCR2 signaling pathway as a regulator of leukocyte adhesion and transmigration through the endothelial cell monolayer.

## 2 Methods

### 2.1 Cell line and culture methods

Primary human neonatal dermal microvascular cells (HNDMVECs, Cat #CC-2516) were purchased from Lonza (Walkersville, MD) and cultured in endothelial cell growth medium MV2 (PromoCell, Heidelberg, Germany, Cat #C-22121) and 100 U/ml penicillin-streptomycin (Gibco, Waltham, MA, Cat #15140122). A HEK293T cell line was purchased from American Type Culture Collection (ATCC) (Manassas, VA, Cat #CRL-3216, RRID: CVCL_0063) and cultured in Dulbecco's Modified Eagle Medium (DMEM) (Gibco, Waltham, MA, Cat # 11995065) with 10% fetal bovine serum (FBS) (Atlanta Biologicals, Flowery Branch, GA, Cat # S11150) and 100 U/ml penicillin-streptomycin. The medium was changed every 2 days, and the cells were harvested by trypsinization. The human acute leukemia monocytic cell line THP-1 (Cat # TIB-202, RRID: CVCL_0006) was purchased from ATCC and cultured in Roswell Park Memorial Institute (RPMI) 1640 medium (ATCC Modification) (Gibco, Waltham, MA, Cat # A1049101) with 10% FBS. The medium was changed every 2 days. Human peripheral blood mononuclear cells (hPBMCs) were purchased from Lonza (Walkersville, MD, Cat # CC-2702) and maintained in RPMI 1640 medium (Gibco, Waltham, MA, Cat # 11875093) with 10% FBS).

### 2.2 Lentiviral production and transduction

A THP-1 cell line that expresses GFP (G-THP-1) was generated through lentiviral transduction. Vectors that contain plasmids of pSL3 (vesicular stomatitis virus G envelope), pSL4 (HIV-1 gag/pol packing genes), pSL5 (rev gene required for HIV-1 envelope protein expression) were a gift from Dr. Murry (University of Washington, Seattle, WA). The plasmid vector pCDH-EF1*α*-MCS*-T2A-GFP was purchased from System Biosciences (Palo Alto, CA, Cat # CD526A-1). The lentiviral vector was packaged in HEK293T cells as previously described (28). Briefly, 3 × 10^6^ of HEK293T cells were seeded in 10-cm dishes to reach 60% confluence in DMEM with 10% FBS and 100 U/ml penicillin-streptomycin. The culture media was changed prior to transfection. A total of 20 μg plasmid DNA (7.5 μg pCDH-EF1-MCS-T2A-copGFP, 2.5 μg pSL3, 6.7 μg pSL4, 3.3 μg pSL5) and 24 μl of lipofectamine 2000 (Thermofisher, Waltham, MA, Cat # 11668019) dissolved in 1,600 μl Opti-MEM I Reduced Serum Medium (Thermofisher, Waltham, MA, Cat # 31985062) were used for the transfection of one dish. The media was replaced after incubating for 6 h at 37 °C with 5% CO_2_. The virus supernatant was collected at 48 h from the media and filtered through a 0.45 µm filter. The media containing the virus was applied to the THP-1 cells at a multiplicity of infection of 6 while spinning at 800xg for 30 min. The transduced G-THP-1 were allowed to expand and sort for GFP expression using a BD Aria III cell sorter to obtain over 92% transduction efficiency.

### 2.3 siRNA transfection

HNDMVECs were seeded on 6-well plates at 5,000 cells/cm^2^ and cultured for 7 days to form a confluent monolayer. Lipofectamine RNAiMAX Transfection Reagent (Thermofisher, Waltham, MA, Cat # 13778150) was used to prepare liposomes with the IL-8 siRNA (Thermofisher, Waltham, MA, s7327, Cat # 4390824) or negative control RNA (Thermofisher, Waltham, MA, Cat # 4404021) according to the manufacturer's instruction. Both the transfection reagent and siRNAs were diluted and mixed to form a siRNA-lipid complex in OptiMEM I Reduced Serum Medium. HNDMVECs were incubated with the siRNA-lipid complex for 2 days at 37 °C. To demonstrate that IL-8 expression was silenced, HNDMVECs were treated with 10 ng/ml recombinant human TNF-α (R&D Systems, Minneapolis, MN, Cat # 210-TA) for 1 h. Then, the IL-8 expression in the HNDMVECs was measured by quantitative real-time PCR.

### 2.4 Cell adhesion assay

HNDMVECs were seeded on 24-well plates at 5,000 cells/cm^2^ and cultured 7 days to form a confluent monolayer. Monocytes or hPBMCs were sheared as previously described (19). Briefly, G-THP-1 cells or hPBMCs at a density of 2 million cells/ml were sheared in a 10-foot-long Masterflex Tygon E-3603 L/S13 pump tubing (Cole-Parmer, Vernon Hills, IL, Cat # MK-06509-13), using a Masterflex miniflex pump model 115/230 VAC 07525-20 (Cole-Parmer, Vernon Hills, IL, Cat # MK-07525-20) at 10 ml/min for 2 h. The tubing was mostly submerged in a water bath with temperature control at 37 °C. At the end of the shear, the G-THP-1 cells or hPBMCs were collected, spun down, and resuspended in fresh media. As a control, G-THP-1 cells or hPBMCs were incubated in a polyvinylchloride (PVC) flask at a density of 2 million cells/ml for 2 h. For the pretreatment groups, HNDMVECs were either incubated with 5 nM of Reparixin (Sigma-Aldrich, St. Louis, MO, Cat # SML2655-5MG) for 30 min or 200 ng/ml of recombinant human IL-8 (R&D Systems, Minneapolis, MN, Cat # 208-IL-010/CF) for 1 h. G-THP-1 cells or hPBMCs, sheared or statically incubated, were then added to a monolayer of HNDMVECs (1 × 10^6^ cells per well in a 24-well plate), followed by a one-hour incubation. Nonadherent G-THP-1 cells or hPBMCs were gently washed twice with sterile phosphate-buffered saline (PBS). The adhered G-THP-1 cells were visualized using a Nikon Eclipse TE200 microscope (Tokyo, Japan) and a Photometric CoolSNAP MYO camera (Tucson, AZ) with a GFP filter. The adhered hPBMCs were visualized with the same microscope and camera with brightfield imaging. The experiments were run in triplicate. The number of adherent G-THP-1 cells or hPBMCs was counted in five randomly selected visible fields and quantified using ImageJ (29) (RRID:SCR_003070).

### 2.5 THP-1 transmigration assay

HNDMVECs were seeded in the upper chamber of a transwell tissue culture insert (6.5 mm diameter, 8 μm pore size polycarbonate membrane; Corning, NY, Cat # 3422) at 5,000 cells/cm^2^ and cultured for 7 days to form a confluent monolayer. Sheared and statically incubated G-THP-1 cells were then added to the upper chamber (2 × 10^5^ cells/well) with the lower chamber filled with RPMI 1640 media. After 24 h of incubation, the upper chamber was removed and the THP-1 cells in the lower chamber were visualized using a Nikon Eclipse TE200 microscope and a Photometric CoolSNAP MYO camera and quantified with ImageJ. The ability of transendothelial migration was determined by counting the migrated G-THP-1 cells in the lower chamber (30). The experiment was run in triplicate. The number of adherent G-THP-1 cells was counted in five randomly selected visible fields and quantified using ImageJ (29).

### 2.6 Immunofluorescent staining

HNDMVECs were seeded on 8-chamber slides (Corning, NY, Cat # 354118) at 5,000 cells/cm^2^ and cultured 7 days to form a confluent monolayer. The sheared or statically incubated G-THP-1 cells were then added to a monolayer of HNDMVECs (1 × 10^6^ cells per well), followed by incubation for 6 h. The cell co-cultures were washed twice with sterile PBS and fixed with formalin. For staining, the fixed cells were permeabilized with 0.1% Triton-X-100 for 10 min, blocked with 1% BSA and human BD Fc block (BD Bioscience, Franklin Lakes, NJ, Cat # 564219) for 1 h, followed by incubation with 1:200 vascular endothelial cadherin (VE-cadherin) monoclonal antibody (16B1) Biotin Conjugate (Thermofisher, Waltham, MA, Cat # 13-1449-82, RRID: AB_466611) for 1 h, 1:500 streptavidin Alexa Fluor 594 Conjugate (Thermofisher, Waltham, MA, Cat # S11227) for 45 min. Nuclei were stained with DAPI. The slides were then washed, mounted, and imaged using a Leica DMI6000 microscope (RRID:SCR_018713) with Leica SP8X confocal. VE-cadherin staining was quantified using ImageJ (29) (RRID:SCR_003070). A minimum of three biological replicas were used for each quantification and a minimum of 6 areas per staining. For VE-Cadherin staining, images were segmented, and a threshold was set on the positive control (no THP1 endothelial cells, Figure 2C). Measurement of the treated conditions was done using the same positive control threshold.

### 2.7 Rhodamine-Phalloidin labeling

HNDMVECs were seeded on 8-chamber slides at 5,000 cells/cm^2^ and cultured for 7 days to form a confluent monolayer. The sheared or statically incubated G-THP-1 cells were then added to a monolayer of HNDMVECs (1 × 10^6^ cells per well), respectively, followed by incubation for 6 h. The cell co-cultures were washed twice with sterile PBS, fixed with formalin, permeabilized with 0.1% Triton-X-100, and blocked with 1% BSA. The cells were then incubated with Rhodamine Phalloidin (ThermoFisher, Waltham, MA, Cat # R415) for 20 min at room temperature, stained with DAPI, washed, mounted, and imaged using a Leica DMI6000 microscope (RRID:SCR_018713) with Leica SP8X confocal. The intercellular gap area was quantified using ImageJ on the slides stained by the Rhodamine Phalloidin (29) (RRID:SCR_003070). The same threshold strategy as for VE-cadherin we used. Further, segmented images were converted to binary images, and a mask was added to the binary image to then create regions of interest (ROIs). The total area of the ROIs was then calculated.

### 2.8 Enzyme-linked immunosorbent assays (ELISA)

The media supernatants of the HNDMVECs and sheared or statically incubated THP-1 cells were collected at 0.5, 1, 3, and 6 h into the co-culture. The levels of IL-1β, IL-6, IL-8, and TNF-α were measured using ELISA kits from Invitrogen (Cat # BMS224-2, 88-7066-88, 88-8086-88, BMS223-4, Waltham, MA). Experiments were run in both biological and technical triplicate.

### 2.9 Quantitative real-time PCR

HNDMVECs were treated with human recombinant IL-8 for 1 h, and total RNA was isolated using the RNeasy Mini Kit (Qiagen, Hilden, Germany, Cat # 74104) and RNase-Free DNAse Set (Qiagen, Hilden, Germany, Cat # 79254). Total RNA (150 ng) was used for cDNA synthesis using the Omniscript RT Kit (Qiagen, Hilden, Germany, Cat # 205113). Quantitative real-time PCR was performed on a QuantStudio 6 Pro real-time PCR system (Thermofisher, Waltham, MA, Cat # A43180) and TaqMan Universal PCR Master Mix (Thermofisher, Waltham, MA, Cat # 4304437) according to the manufacturer instructions. Both biological and technical triplicate were run. TaqMan primers of selectin E (Hs00174057_m1, Cat # 4331182), intercellular adhesion molecule 1 (ICAM1) (Hs00164932_m1, Cat # 4331182) and vascular cell adhesion molecule 1 (VCAM1) (Hs01003372_m1, Cat # 4331182) were purchased from Thermofisher (Waltham, MA). To demonstrate that IL-8 expression was silenced, HNDMVECs were treated with 10 ng/ml TNF-α (R&D Systems, Minneapolis, MN, Cat # 210-TA) for 1 h, and the IL-8 expression was measured. The same preparation was done to perform quantitative real-time PCR. Taqman primer of IL-8 (Hs00174103_m1, Cat # 4331182) was purchased from Thermofisher (Waltham, MA).

### 2.10 Flow cytometry

Cells were with 4% PFA (Electron Microscopy Sciences, Hatfield, USA) and then stained with primary antibodies against CD62E, E-Selcetin (PE-conjugated) (# 12-0627-42, Thermofisher, Walthman, MA), CD54, ICAM-1 (PE-conjugated) (#12-0549-42, Thermofisher, Waltham, MA), and CD106, VCAM-1 (#12-1059-42, Thermofisher, Waltham, MA) at 4 °C for 1 h in PBS supplemented with 0.09% (w/v) sodium azide (ThermoFisher, Waltham, USA) and 1% heat-inactivated FBS. Gating was performed using isotype controls following singlets and live/dead gating. Data were acquired using a BD FACSymphony A3 (BD, Franklin Lakes, USA) and analyzed with FlowJo 10 (BD, Franklin Lakes, USA).

### 2.11 Statistical analysis

All experiments were run in triplicate. All quantitative data were expressed as mean ± standard deviation within groups. Pairwise comparisons between groups were conducted using the ANOVA test and Tukey's *post-hoc* test. Statistical significance is denoted by “*”. *P* values less than 0.05 are indicated by a single symbol and *P* values less than 0.01 are indicated by double symbols.

## 3 Results

### 3.1 CPB shear promotes mononuclear cell adhesion to and transmigration through the endothelial monolayer

**Figure 1:**
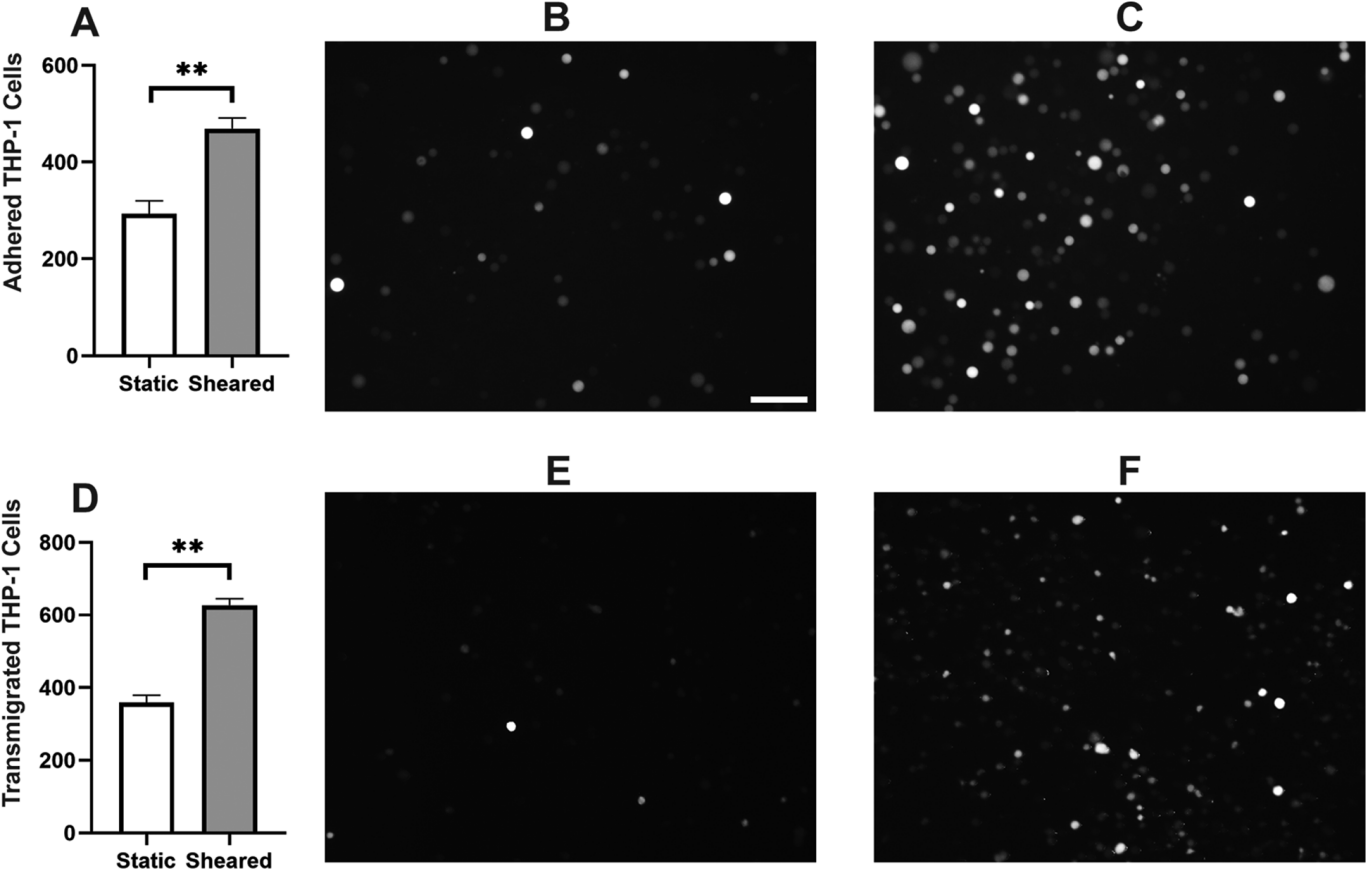
For cell adhesion assay, G-THP-1 cells were sheared in the *in vitro* CPB circuit for 2 h or cultured statically in a PVC flask, followed by co-culture with confluent HNDMVECs for 1 h. At the end of the co-culture, non-adherent G-THP-1 cells were rinsed, and adherent G-THP-1 cells were quantified. **(A)** Quantitative analysis of adhered G-THP-1 cells on the endothelial cell monolayer. **(B)** Adhesion of G-THP-1 cells incubated statically in a PVC flask. **(C)** Adhesion of G-THP-1 cells sheared in a CPB circuit. For transmigration assay, G-THP-1 cells were sheared in the *in vitro* CPB circuit for 2 h or cultured statically in a PVC flask. The G-THP-1 cells were then added to confluent HNDMVECs grown on the top chamber of the transwell insert. After an overnight incubation, the G-THP-1 cells transmigrated to the bottom chambers were quantified. **(D)** Quantitative analysis of transmigrated THP-1 cells through endothelial cell monolayer. **(E)** Transmigrated G-THP-1 cells incubated statically in a PVC flask. **(F)** Transmigration of G-THP-1 cells sheared in a CPB circuit. Scale bar = 100 μm. For additional images see Supplementary Figure S2. All experiments were run in triplicate. All quantitative data were expressed as mean ± standard deviation within groups. Pairwise comparisons between groups were conducted using the ANOVA test and Tukey's *post-hoc* test. Statistical significance is denoted by “*”. *P* values less than 0.05 are indicated by single symbols and *P* values less than 0.01 are indicated by double symbols.

Monocyte infiltration is commonly observed in the inflammatory response in CPB (31–33). During this process, activated monocytes adhere to the vascular wall, transmigrate through the intercellular tight junctions, and reach different organs (34–37). To characterize the response of monocytes specifically to CPB shear, we sheared the THP-1 cells in the CPB circuit, co-cultured them with confluent HNDMVECs for 1 h, and quantified the number of adherent THP-1 cells. As shown in Figure 1A, exposing THP-1 to shear stress resulted in a two-fold increase in THP-1 cells adhering to the HNDMVEC monolayer. A similar trend was observed for PBMCs (Supplementary Figure S1). Furthermore, significantly more sheared THP-1 cells were observed in the bottom chamber of the transwells that had been seeded with HNDMVECs compared with static THP-1 cells (Figure 1D). These results suggest that exposure to CPB shear-induced monocytic cells to be adherent to and to transmigrate through a monolayer of endothelial cells.

### 3.2 The interaction of sheared THP-1 cells with HNDMVECs results in disrupted intercellular junction

**Figure 2:**
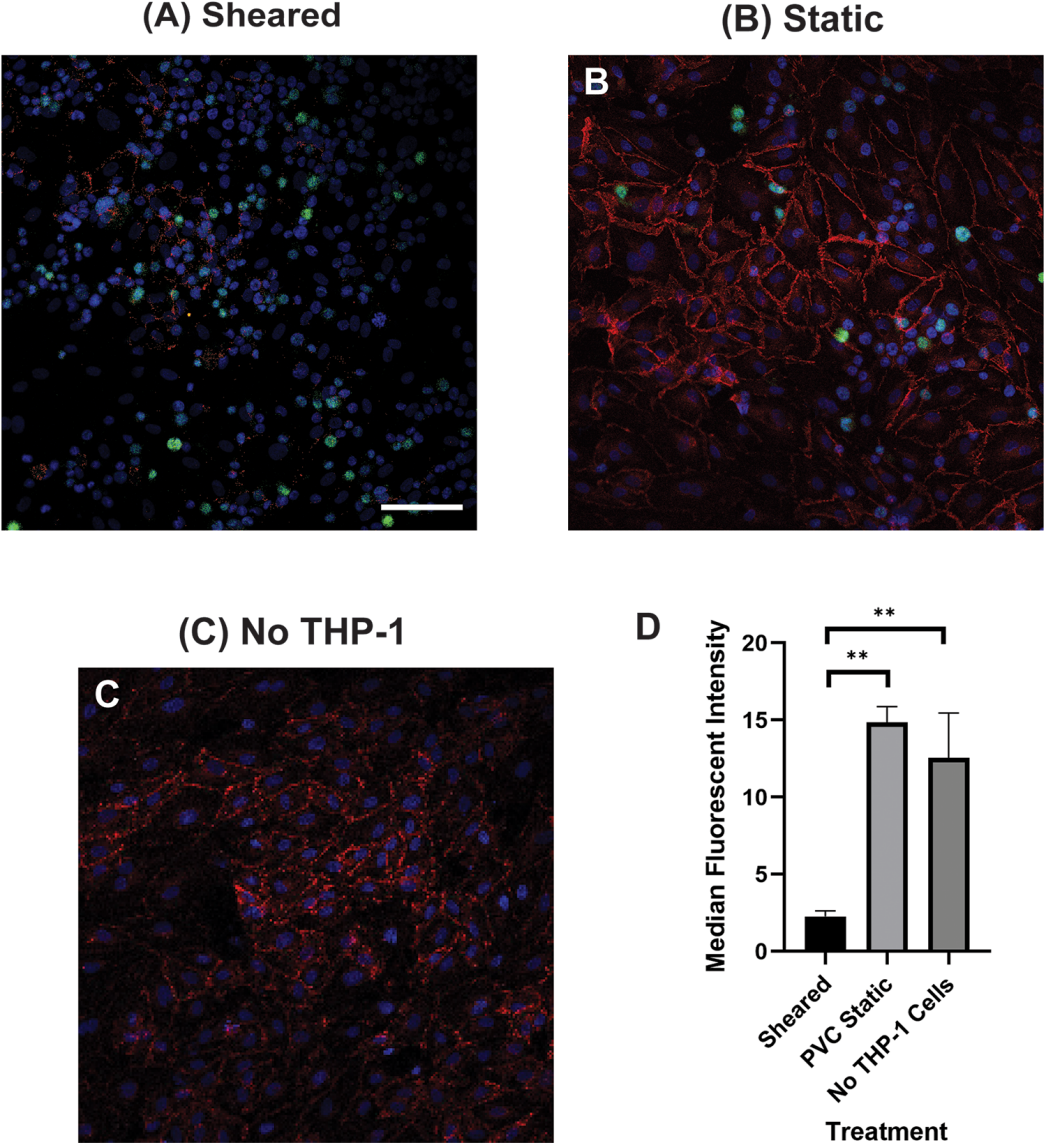
VE-cadherin of HNDMVECs co-cultured with **(A)** sheared THP-1 cells, **(B)** THP-1 cells statically incubated in PVC flask, and **(C)** No THP-1 treatment, visualized by immunofluorescent staining. For additional images see Supplementary Figure S3. Red = VE-Cadherin, Green = THP-1 Cells, Blue = DAPI. Scale bar = 100 μm. **(D)** Quantification of VE cadherin staining. G-THP-1 cells were sheared in the *in vitro* CPB circuit for 2 h or cultured statically in a PVC flask, followed by co-culture with confluent HNDMVECs for 6 h. After the co-culture, the cells were fixed and stained with VE-cadherin antibodies. All experiments were run in triplicate. All quantitative data were expressed as mean ± standard deviation within groups. Pairwise comparisons between groups were conducted using the ANOVA test and Tukey's *post-hoc* test. Statistical significance is denoted by “*”. *P* values less than 0.05 are indicated by single symbols and *P* values less than 0.01 are indicated by double symbols.

**Figure 3:**
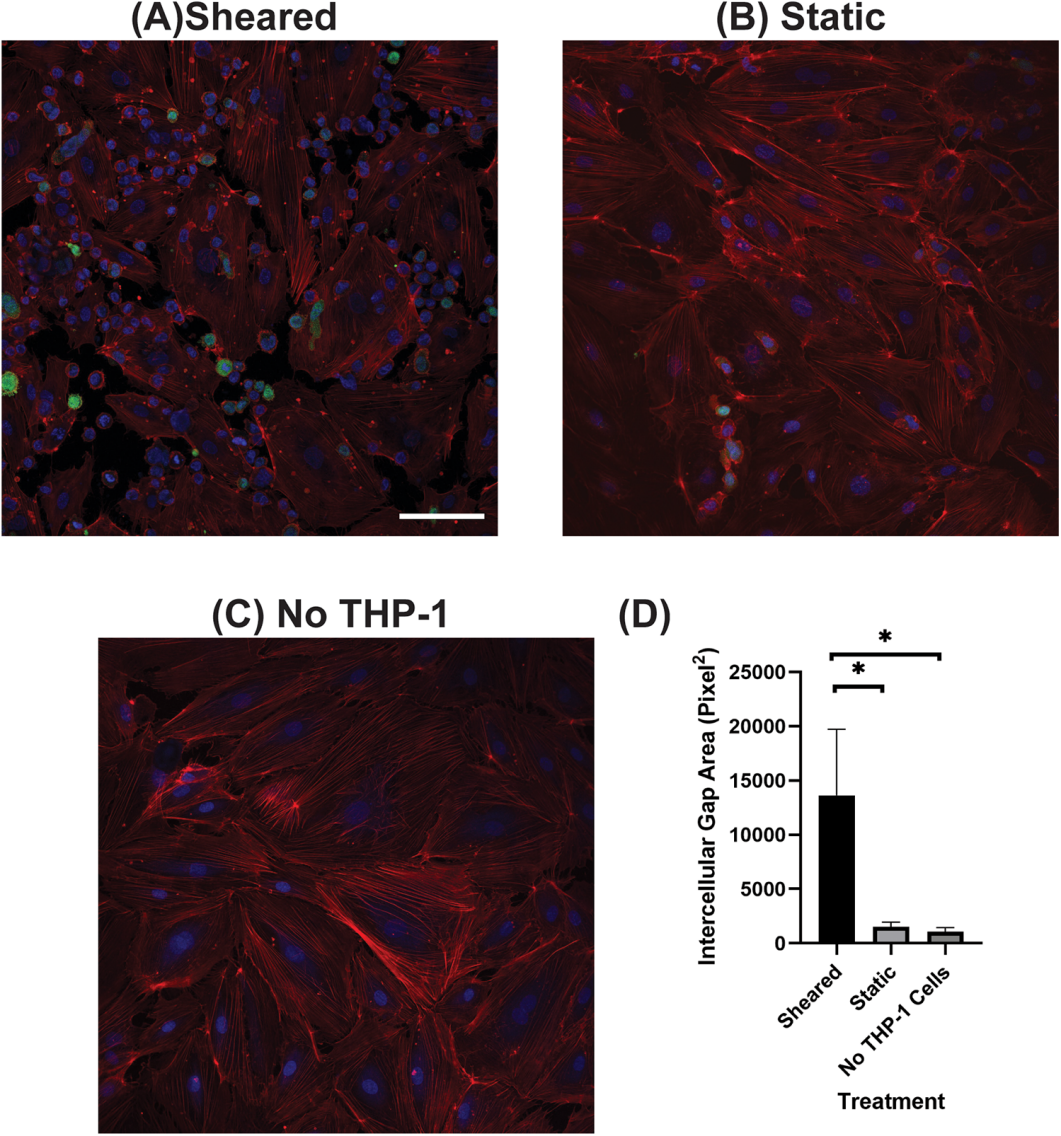
F-actin of HNDMVECs co-cultured with **(A)** sheared THP-1 cells, **(B)** THP-1 cells statically incubated in PVC flask, and **(C)** No THP-1 treatment, stained by rhodamine-phalloidin. For additional images see Supplementary Figure S4. Red = F-actin, Green = THP-1 Cells, Blue = DAPI. Scale bar = 100 μm. **(D)** Quantification of intercellular gap area of endothelial cells. All experiments were run in triplicate. All quantitative data were expressed as mean ± standard deviation within groups. Pairwise comparisons between groups were conducted using the ANOVA test and Tukey's *post-hoc* test. Statistical significance is denoted by “*”. *P* values less than 0.05 are indicated by single symbols and *P* values less than 0.01 are indicated by double symbols.

The increasing transmigration behavior of sheared monocytes prompted us to examine if the sheared cells were disrupting the barrier function in the endothelial monolayer. To examine the intercellular adherens junction and reorganization of the cytoskeleton, HNDMVECs were stained for VE-cadherin and F-Actin after co-culture with sheared or static THP-1 cells. Co-culturing with sheared THP-1 cells resulted in the loss of adherens junction while they remained intact in endothelial cells cultured with static THP-1s (Figures 2A, B). Similarly, the static group showed robust actin filament lining intracellularly (Figure 3B). In the sheared group, we observed that THP-1 cells clustered at the intercellular area (Figure 3A). Further, actin filaments reorganized and the intercellular gap areas between endothelial cells were increased in the sheared group compared to the static group and the endothelial cells without THP-1 treatment. These results imply increasing motility of the endothelial cells in response to the sheared monocyte insult.

### 3.3 The interaction between sheared THP-1 and HNDMVECs results in an increased Il-8 release level

**Figure 4:**
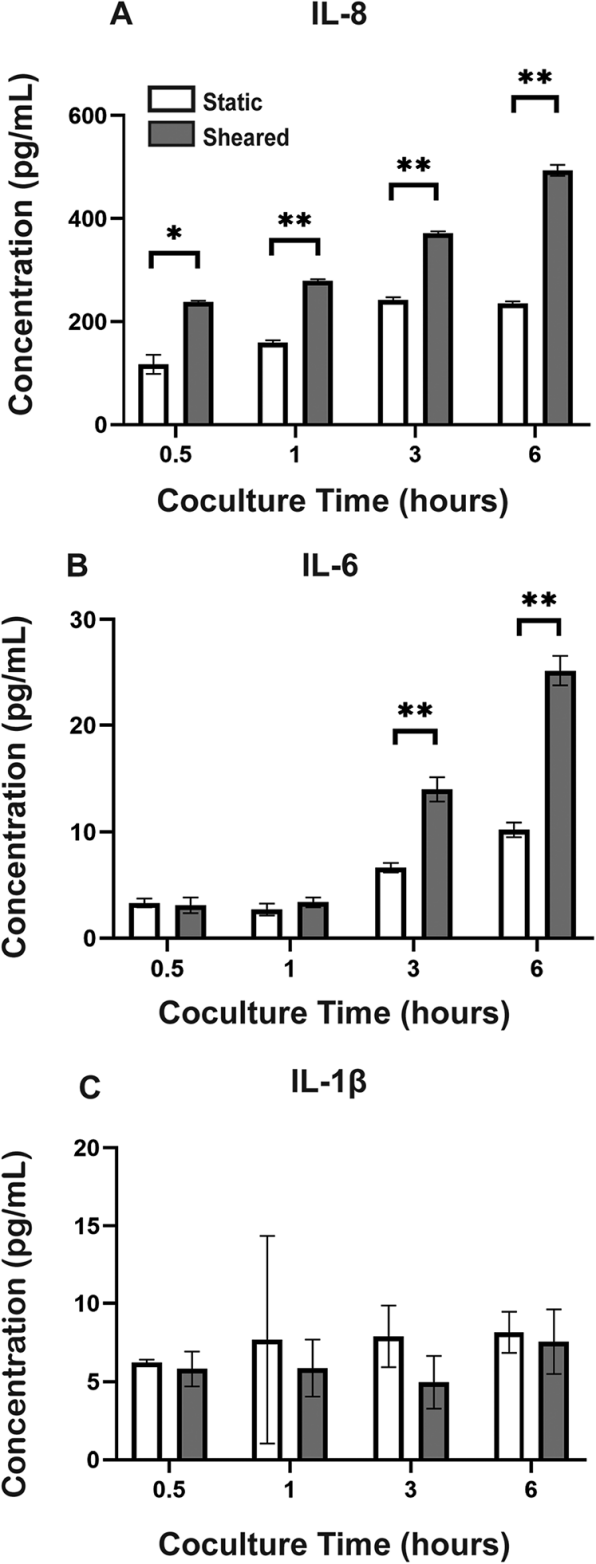
Cytokine levels of **(A)** IL-8, **(B)** IL-6, and **(C)** IL-1β in the co-culture media of sheared or static THP-1 cells and HNDMVECs measured at 0.5, 1, 3, and 6 h. All experiments were run in triplicate. All quantitative data were expressed as mean ± standard deviation within groups. Pairwise comparisons between groups were conducted using the ANOVA test and Tukey's *post-hoc* test. Statistical significance is denoted by “*”. *P* values less than 0.05 are indicated by single symbols and *P* values less than 0.01 are indicated by double symbols.

To analyze the inflammatory cytokines secreted by sheared or static THP-1 cells and HNDMVECs, we collected co-culture media at 0.5, 1, 3, and 6 h and measured the levels of IL-1β, IL-6, IL-8, and TNF-α using ELISA. The sheared group released significantly more IL-8 than the static group at every time point (Figure 4A). The level of IL-8 increased over the period of 6 h in the shear group while it leveled off at 3 h in the static group (Figure 4A). IL-6 level in the sheared group significantly increased at 3 and 6 h but the concentration was about 20-fold lower than IL-8 (Figure 4B). No significant release of IL-1β and TNF-α was detected throughout the co-culture (Figure 4C, TNF-α data not shown). These findings indicate that treating HNDMVECs with sheared THP-1 specifically induced IL-8 release.

### 3.4 Reparixin, a CXCR2 antagonist, inhibits the adhesion of sheared THP-1 cells while IL-8 promotes the adhesion of untreated THP-1 cells

**Figure 5:**
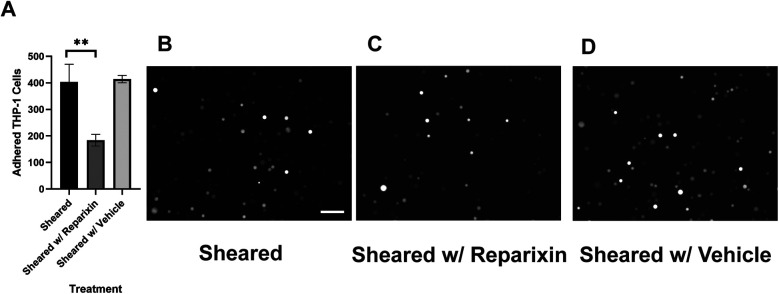
**(A)** quantitative analysis of sheared G-THP-1 cells adhering to **(B)** untreated HNDMVECs, **(C)** HNDMVECs pretreated with 5nM reparixin for 30 min, and **(D)** HNDMVECs pretreated with PBS. Scale bar = 100 μm. For additional images see Supplementary Figure S6. All experiments were run in triplicate. All quantitative data were expressed as mean ± standard deviation within groups. Pairwise comparisons between groups were conducted using the ANOVA test and Tukey's *post-hoc* test. Statistical significance is denoted by “*”. *P* values less than 0.05 are indicated by single symbols and *P* values less than 0.01 are indicated by double symbols.

**Figure 6:**
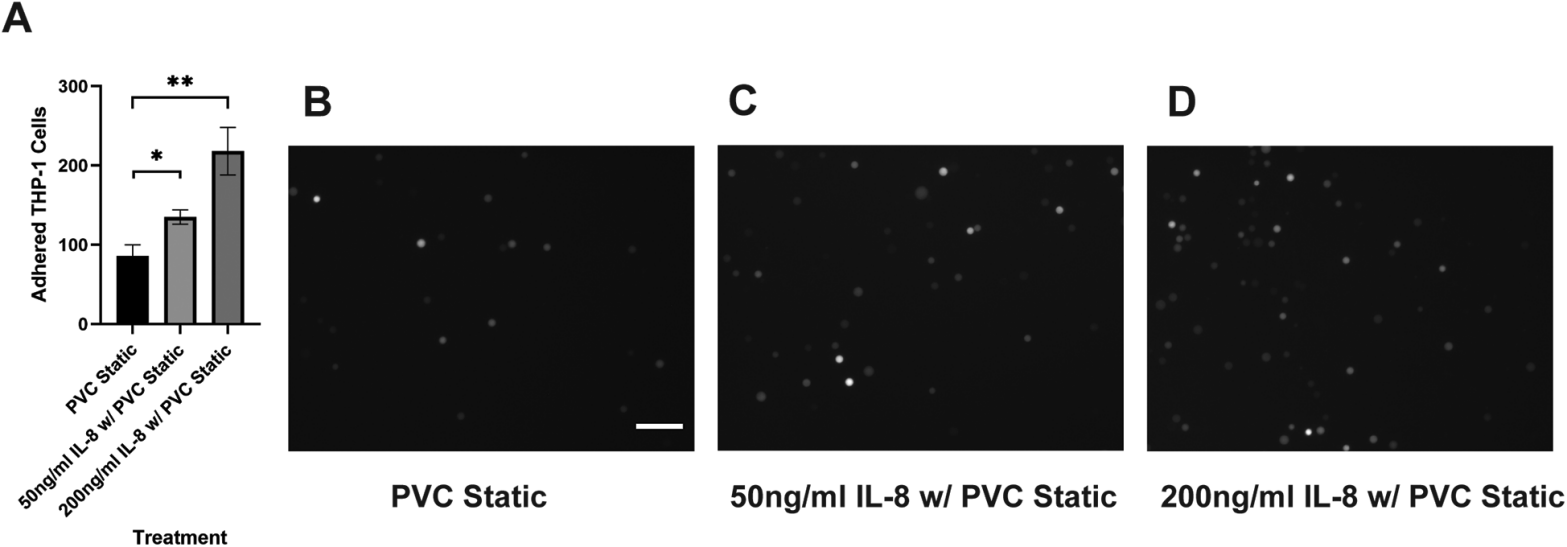
**(A)** quantitative analysis of statically incubated G-THP-1 cells adhering to **(B)** untreated HNDMVECs, **(C)** HNDMVECs pretreated with 50 ng/ml IL-8 for 1 h, and **(D)** HNDMVECs pretreated with 200 ng/ml IL-8 for 1 h. For additional images see Supplementary Figure S7. Scale bar = 100 μm. All experiments were run in triplicate. All quantitative data were expressed as mean ± standard deviation within groups. Pairwise comparisons between groups were conducted using the ANOVA test and Tukey's *post-hoc* test. Statistical significance is denoted by “*”. *P* values less than 0.05 are indicated by single symbols and *P* values less than 0.01 are indicated by double symbols.

Our results suggested that the adhesion of THP-1 cells on the endothelial cell is positively correlated to the IL-8 release during co-culture. To further investigate the role of IL-8 in mediating the interaction between THP-1 cells and the endothelial cells, we treated the endothelial cells with Reparixin, an inhibitor of the IL-8 receptors, CXCR1/2, and then co-cultured with sheared THP-1 cells (CXCR2 staining of HNDMVECs see Supplementary Figure S5). Reparixin treatment reduced the adhesion of sheared THP-1 similar to static control (Figure 5C). When we pre-treated HNDMVECs with 50 and 200 ng/ml IL-8, the adhesion of untreated THP-1 cells was promoted in a dose-dependent manner (Figures 6C, D). These results suggested that blocking IL-8 signaling through the CXCRs prevented the adhesion of shear-activated THP-1 cells.

### 3.5 Silencing IL-8 expression in endothelial cells reduced the adhesion of sheared monocytes

**Figure 7:**
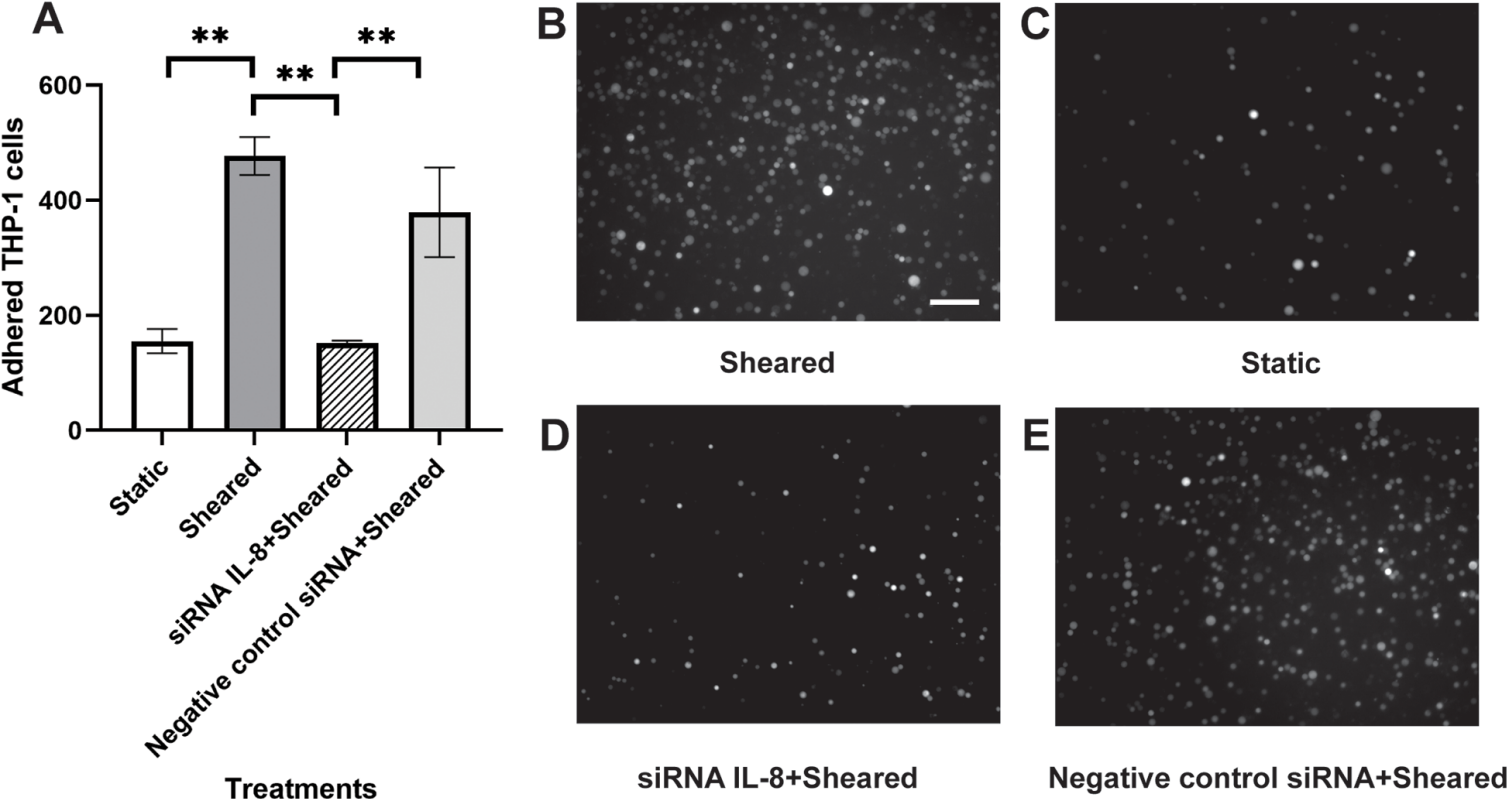
**(A)** quantitative analysis of **(B)** statically incubated G-THP-1 cells adhering to HNDMVECs, and sheared G-THP-1 cells adhering to **(C)** HNDMVECs, **(D)** HNDMVECs treated with IL-8 siRNA, and **(E)** HNDMVECs treated with negative control siRNA. For additional images see Supplementary Figure S9. Scale bar = 100 μm. All experiments were run in triplicate. All quantitative data were expressed as mean ± standard deviation within groups. Pairwise comparisons between groups were conducted using ANOVA test and Tukey's *post-hoc* test. Statistical significance is denoted by “*”. *P* values less than 0.05 are indicated by single symbols and *P* values less than 0.01 are indicated by double symbols.

To further identify the source of IL-8 in the co-culture, we silenced the IL-8 expression in the HNDMVECs with siRNA. HNDMVECs were incubated with the lipid-siRNA complex for 2 days and then stimulated with 10 ng/ml TNF-α for 1 h. Results from qPCR showed significant upregulation of IL-8 expression in the group not treated with siRNA or treated with scramble siRNA, while the IL-8 expression in the group treated with siRNA remained low, suggesting IL-8 expression in HNDMVECs was silenced (Supplementary Figure S8). The HNDMVECs were then co-cultured with sheared THP-1 cells and adhered cells were quantified after 1 h. Adhesion of THP-1 cells was significantly reduced in the group treated with the siRNA compared with the group treated with scramble siRNA or without treatment (Figure 7). These results indicate that endothelial-derived IL-8 largely contributes to the increased adhesion of sheared THP-1.

### 3.6 IL-8 upregulated adhesion molecules on the endothelial cells

**Figure 8:**
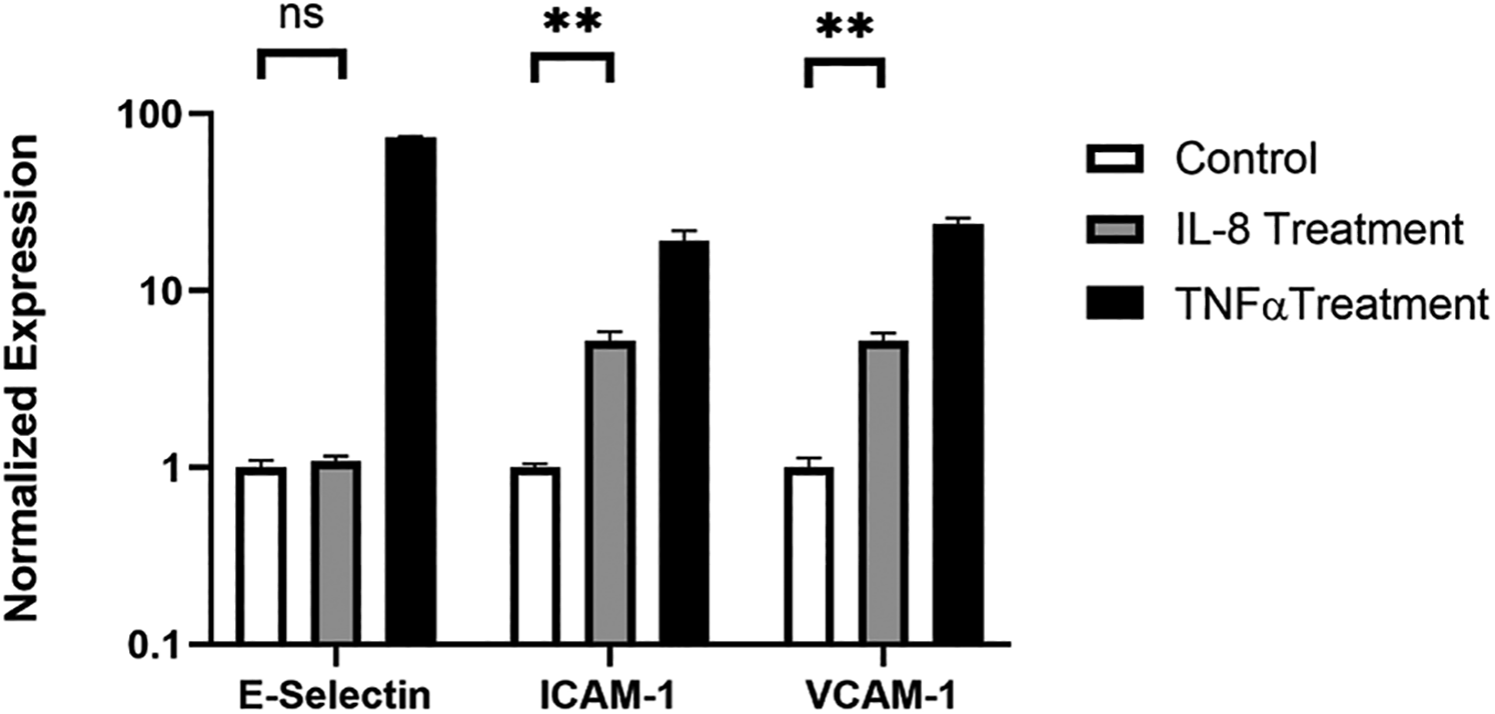
Expression of e-selectin, ICAM1, and VCAM1 in HNDMVECs treated with IL-8, TNF-α, and control measured by RT-qPCR. All experiments were run in triplicate. All quantitative data were expressed as mean ± standard deviation within groups. Pairwise comparisons between groups were conducted using ANOVA test and Tukey's *post-hoc* test. Statistical significance is denoted by “*”. *P* values less than 0.05 are indicated by single symbol and *P* values less than 0.01 are indicated by double symbols.

To gain mechanistic insight into how IL-8 promotes mononuclear cell adherence to endothelial cells, we examined the expression of adherence molecules in endothelial cells. qPCR and FACS analysis were performed to examine the expression of adhesion molecules, including e-selectin, VCAM1, and ICAM1. HNDMVECs were treated with IL-8 with TNF-α treatment being a positive control to stimulate the endothelial cells. All three adhesion molecules were upregulated in the positive control group (Figure 8 and Supplementary Figure S10). In the IL-8 group, there was a 5-fold increase in both the expressions of ICAM1 and VCAM1, while the expression of e-selectin remained unchanged (Figure 8). These results indicate that the increasing adhesion of sheared THP-1 cells was likely to be mediated by the IL-8 signaling pathway through upregulated adhesion molecule expression on the endothelial cells.

## 4 Discussion

**Figure 9:**
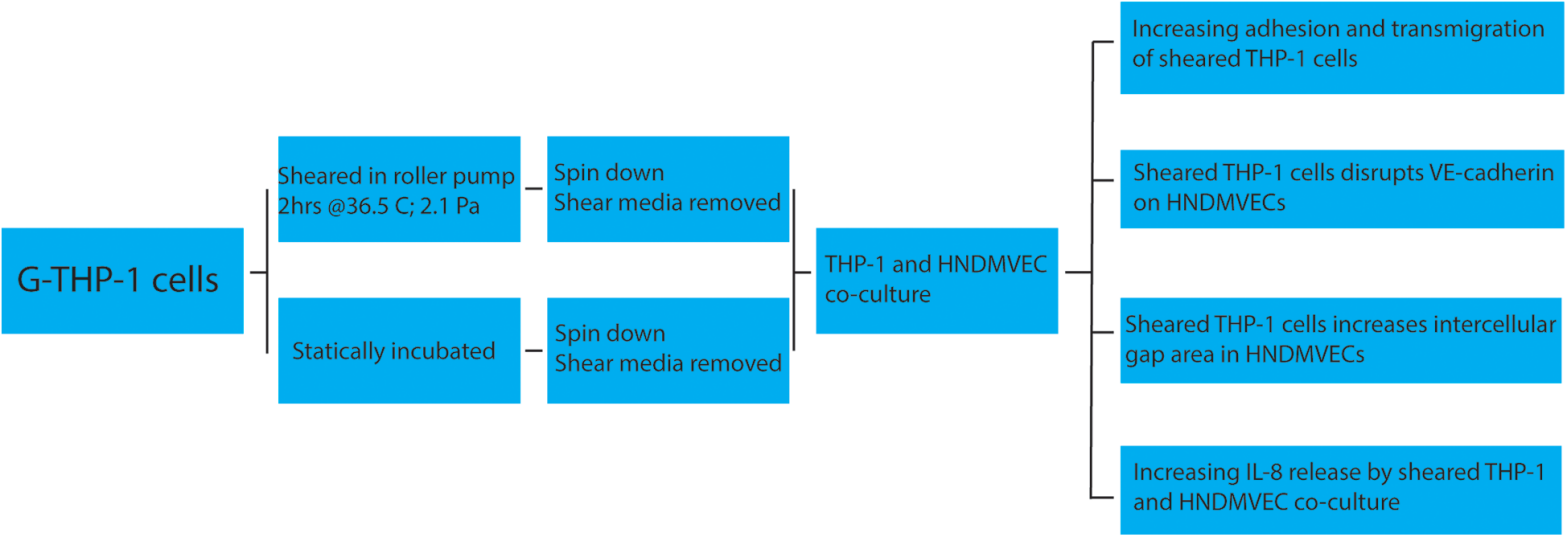
Cartoon describing a proposed mechanism of shear stress activation THP1 effect on endothelial cells.

With this study, we are, to our knowledge, the first to show that CPB shear stress-activated monocytes adhere to and migrate through an intact and unstimulated endothelial monolayer. We used an *in vitro* CPB model to probe the interaction between shear stress-activated THP-1 monocytes and quiescent confluent HNDMECs. Our data demonstrated that an average CPB shear stress of 2.1 Pa or 21 dyne/cm^2^ promoted the adhesion and transmigration of monocytes to and through the endothelial monolayer. This was accompanied by the disruption of the adherens junction and reorganization of the cytoskeleton in the endothelial cells (diagram of the experimental setup and results see Figure 9). IL-8, which was significantly upregulated in the co-culture media, played a pivotal role in mediating the crosstalk between monocytes and endothelial cells. Inhibiting the binding of IL-8 to the CXCRs on endothelial cells prevented sheared monocytes from adhering. Preincubating endothelial cells with IL-8 resulted in increasing adhesion of untreated monocytes, suggesting endothelial cell activation. We further established that endothelial-derived IL-8 was responsible for the sheared-monocyte adhesion. Finally, IL-8 also induced ICAM1 and VCAM1 expression in HNDMVECs. This suggests that IL-8 may promote the adhesion of the CPB shear stress-treated monocytes by activating a subset of adhesion molecules on the microvascular endothelial cells.

The sequela of leukocyte rolling and binding to activated endothelial cells and transmigration through the endothelial monolayer is a fundamental process of inflammation responding to mediators produced by a traumatic injury. We have recently shown that using *in vitro* CPB setup recapitulating a prolonged CPB surgery, monocytes circulating for 2 h at 21 dyne/cm^2^ showed increased expression of IL-8 and TNF-α (19). Now we also find that these cells can adhere to and transmigrate through an endothelial monolayer without additional stimulation. These results suggest that CPB shear stress is sufficient to promote monocyte adhesion and transmigration. Additionally, loss of endothelial membrane VE-cadherin staining correlated with the formation of gaps in the monolayer. Interestingly, Baratchi et al., using a microfluidic system modeling the change in shear stress observed in aortic stenosis, recently described calcium flux-dependent activation of monocytes in response to elevated shear stress and their ability to adhere to TNF-α stimulated human umbilical vein endothelial cells (38). Our data strongly suggest that TNF-α was not playing a role in our CPB shear stress-induced THP-1/endothelial cell interactions, as we did not find changes of TNF-α levels in the co-culture medium, even if TNF-α was induced by CPB shear stress in monocytes as shown previously (19). A potential explanation for the absence of TNF-α in the co-culture is that CPB shear stress-activated THP-1 media was refreshed before co-culture with the endothelial monolayer, suggesting that when the shear stress stimulus is removed, the monocytes stop producing TNF-α. These results point to different factors mediating the monocyte-endothelial cell interaction in our system.

We show that the monocyte-endothelial cell interaction was promoted by the addition of IL-8 and inhibited by the CXCR antagonist Reparixin. These results suggest that IL-8 mediates the interaction between monocytes and endothelial cells in CPB shear stress conditions. Accordingly, we have previously shown that IL-8 in the monocytes is directly upregulated by CPB shear stress (19). Here we find that IL-8 was also elevated in the media of CPB shear stress-stimulated THP-1 and endothelial cell co-cultures. The results of the current study revealed that IL-8 is a main cytokine mediating the interaction between monocytes and endothelial cells in a CPB setting. To clarify the cell source of IL-8 in the co-culture system we used siRNA silencing. We showed that silencing the IL-8 expression in the endothelial cells prevented the adhesion of sheared monocytes. This result suggests that the endothelial cells mainly contributed to the IL-8 release during the co-culture. The proposed mechanism of monocyte adhesion is that CPB shear stress-activated THP-1 cells stimulated the IL-8 release from endothelial cells and led to the upregulation of endothelial adhesion molecules. This autocrine signaling of IL-8 has been reported in endothelial cells to support the capillary tube formation and neovascularization in the absence of external IL-8 signaling (39–45). The current study design limitation did not rule out the possible contribution of IL-8 from monocytes knowing that monocytes produce IL-8 (19). It is also possible that THP-1 monocytes continue to produce IL-8 after the CPB shear stress stimulus is removed and thus promote their own adhesion. Interestingly, IL-8 appears to autocrinally promote its own transcription in monocytes but not in neutrophils (46), suggesting a feed-forward loop of self-activation. Future investigation will be needed to evaluate the role of monocyte-derived IL-8 in our system. Apart from the change in IL-8 levels, we observed that the IL-6 level increased significantly at 6 h post-sheared THP-1 cell/endothelial cells co-culture. Although studies have linked IL-6 levels to inflammation and postoperative mortality after CPB (47–49), in our model, significant sheared monocyte adhesion was detected at 1 h into co-culture, prior to IL-6 release, and thus suggesting an IL-6 independent mechanism. In addition, we demonstrate strong inhibition of CPB-induced THP-1 cell adhesion to endothelial monolayer by the CXCR1/2 inhibitor Reparixin and IL-8 siRNA, thus causally implicating the detrimental effect of IL-8 in endothelial function induced by CPB shear stress-activated monocytes, which can further lead to vessel leakage and monocytes tissue invasion.

IL-8 is well-known to regulate neutrophil functions by promoting chemotaxis, causing the release of lysosomal enzymes, upregulation of adhesion molecules, increasing intracellular calcium, and priming of the oxidative burst (50–54). Much less is known about the functions of IL-8 in monocytes. In flow conditions, IL-8 stimulates adhesion to E-selectin-expressing endothelial cells and promotes monocyte polarization toward an M1 pro-inflammatory phenotype (55–57). In our system, it appears that IL-8 promoted endothelial-specific processes facilitating monocyte adhesion that was strongly inhibited by blocking the IL-8 receptors on endothelial cells. Further studies will be necessary to assess the direct effects of IL-8 on CPB shear stress-activated monocytes. In addition, endothelial cells have been shown to express the IL-8 receptors CXCR1 and CXCR2 and respond to IL-8 by activating angiogenesis processes, including proliferation, survival, tube morphogenesis, and MMP production (58). The binding of IL-8 on endothelial cells can induce cytoskeletal reorganization through Rho and Rac signaling pathways in a phosphoinositide 3-kinase-dependent manner (59–61), which are correlated to the clustering of E-selectin, intercellular adhesion molecules, and vascular cell adhesion molecules. IL-8 treatment increases the permeability of the endothelium, likely facilitating the subsequent transmigration of monocytes (24, 59, 62). Further, IL-8 has been shown to activate the NF-*κ*B pathway in several immune cell lines, including THP-1, and to induce the expression of VCAM-1 and ICAM-1 in U937 cells (63). Accordingly, our studies also show that IL-8 is a strong inducer of ICAM1 and VCAM1 RNA expression in the microvascular endothelial cells. We also observed IL-8-dependent endothelial cell cytoskeleton rearrangement and VE-cadherin disruption.

**Figure 10:**
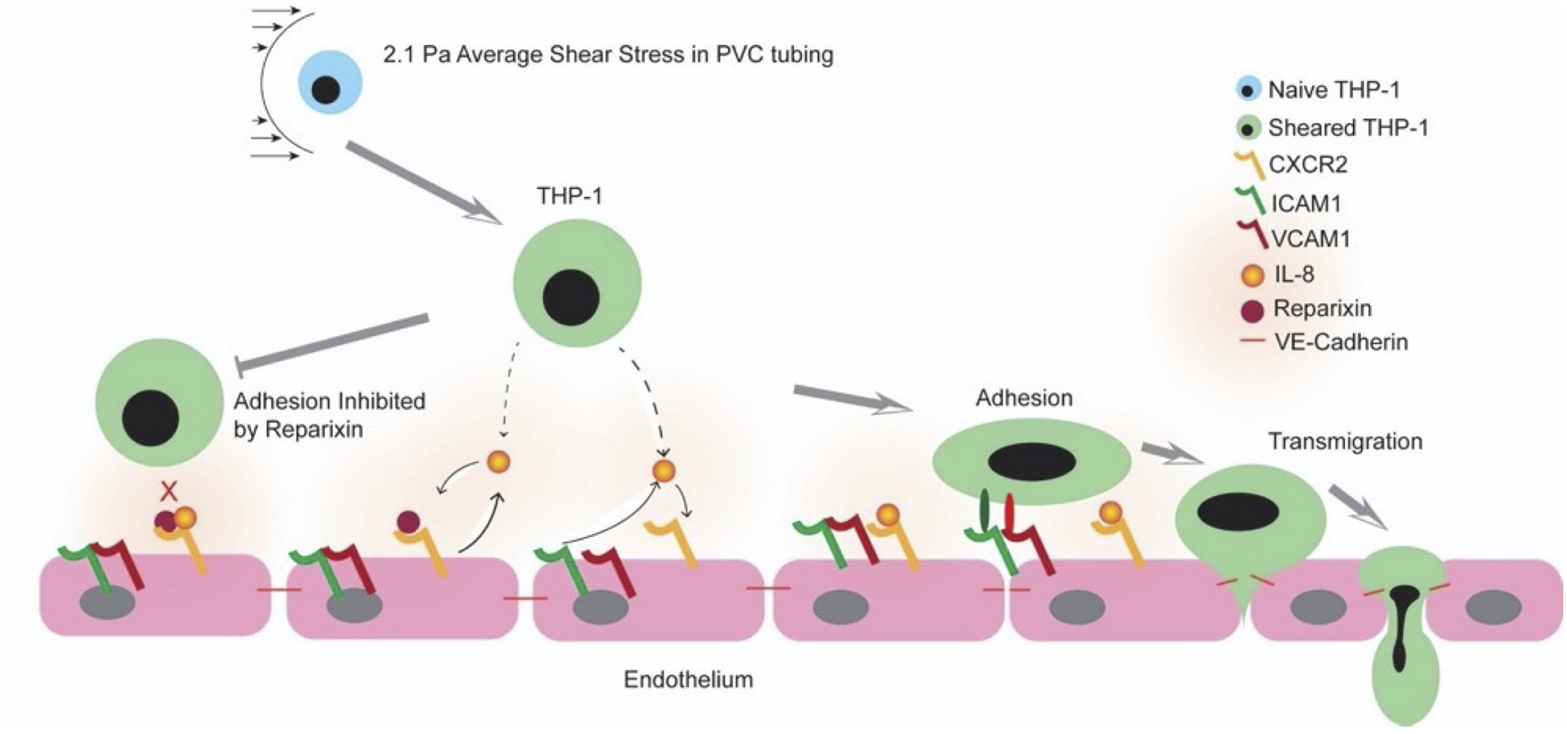
Cartoon representing a working model of the shear stress and IL-8 signaling CPB-induced inflammatory response.

Evidence has shown that inflammation leads to organ damage in CPB patients and that elevated plasma level of IL-8, among other cytokines, positively correlates with acute kidney injury, brain injury in newborns, and intensive care length of stay (23–27). Experimental evidence also indicates CPB-dependent transmigration and tissue infiltration of leukocytes and neutrophils (31). In addition, the inflammatory response to CPB can vary by different ages. Pediatric patients are more susceptible to brain injuries than adults after CPB (64). However, the pathological mechanisms behind organ damage in CPB still remain unresolved. This is the first study to our knowledge to show that CPB shear stress-activated monocytes affected endothelial integrity and promoted monocyte adhesion and transmigration in an IL-8-dependent manner, suggesting that IL-8-mediated signaling pathway may play an important role in the onset of CPB-induced inflammatory response and tissue infiltration at the cellular level (cartoon, Figure 10) These studies further suggest that blocking IL-8 receptors may prove a promising approach to reduce endothelial injury and tissue and organ damage observed in prolong CPB and pediatric patients undergoing CPB.

This study has several limitations. The neonatal dermal endothelial cells used in this study are derived from only one tissue. Indeed, CPB-induced systemic insult involves the endothelium of different organs including the brain, kidney, and lung (65–67). The endothelial cells from different organs are heterogeneous (68, 69) and thus may result in different responses to CPB shear and shear-activated blood cells. For example, it is established that aortic endothelial cells are more susceptible to TNF-α-mediated monocyte adhesion compared to pulmonary microvascular endothelial cells and renal glomerular endothelial cells (70). Therefore, future studies will focus on characterizing the interactions between CPB-affected monocytes and endothelial cells isolated from different organs. In addition, THP-1 cells and endothelial cells were co-cultured in a static condition. Indeed, it is well-established that physiological flow protects endothelial cells from injury (71). The static condition also does not fully recapitulate the flow condition in which the cellular interactions take place during CPB. Future studies will focus on the addition of a flow chamber loaded with endothelial cells to the *in vitro* CPB circuit. This will help the characterization of cellular interactions more accurately.

## Data Availability

The original contributions presented in the study are publicly available. This data can be found here: https://figshare.com/authors/Hao_Zhou/18715732.
