# Supplementary material for: Monocyte adhesion to and transmigration through endothelium following cardiopulmonary bypass shearing is mediated by IL-8 signaling
Source: Front Cardiovasc Med. 2024 Dec 11;11:1454302. doi: 10.3389/fcvm.2024.1454302 (PMC11668754; doi:10.3389/fcvm.2024.1454302)
Supplement: Supplementary file 1 [file Datasheet1.pdf]

Supplementary Material

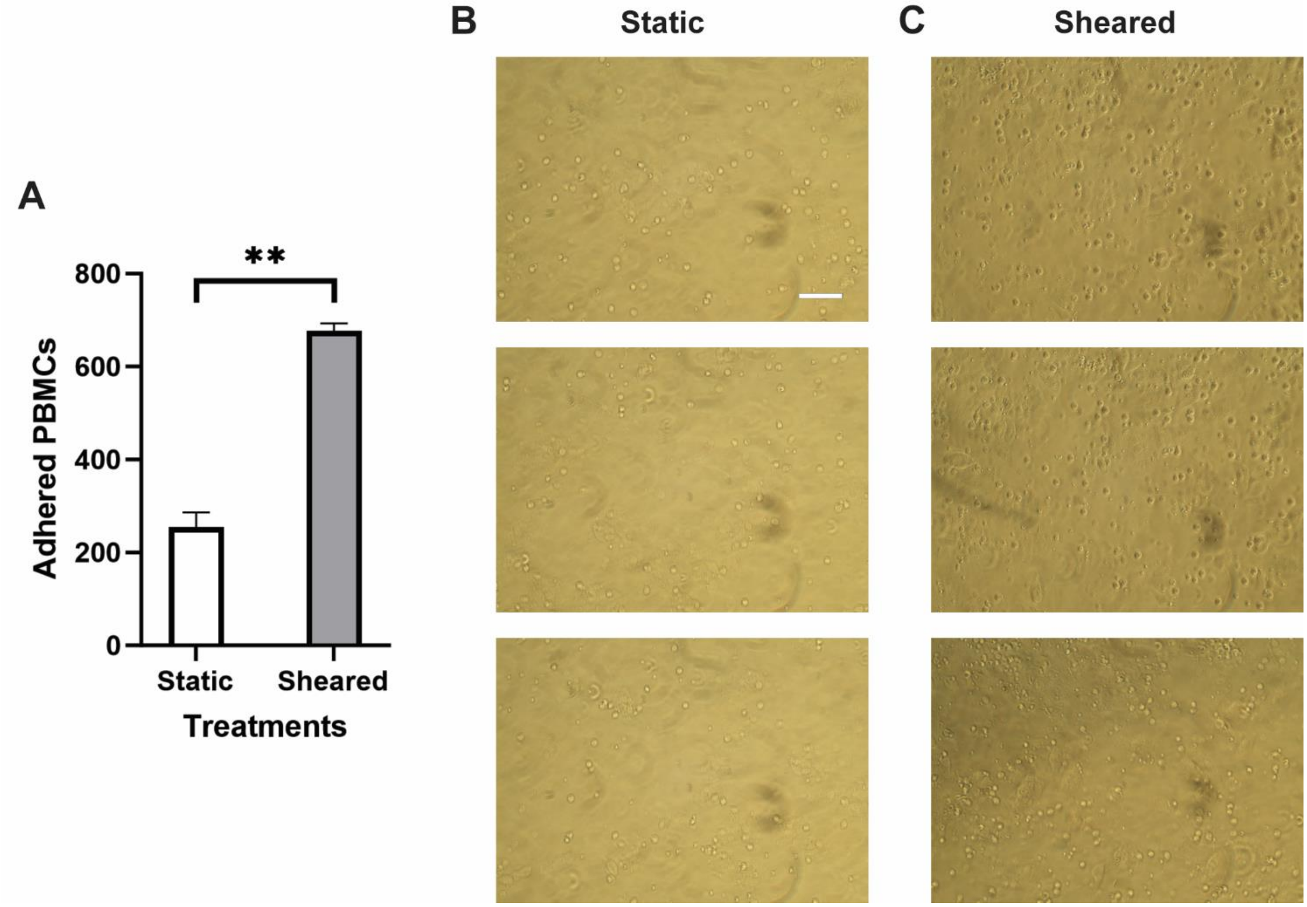

Supplementary Figure 1. (A) Quantitative analysis of adhered PBMCs on the endothelial cell monolayer. **(B)** Adhesion of G-THP-1 cells incubated statically in a PVC flask. **(C)** Adhesion of G-THP-1 cells sheared in a CPB circuit. Scale bar = 100 μm.

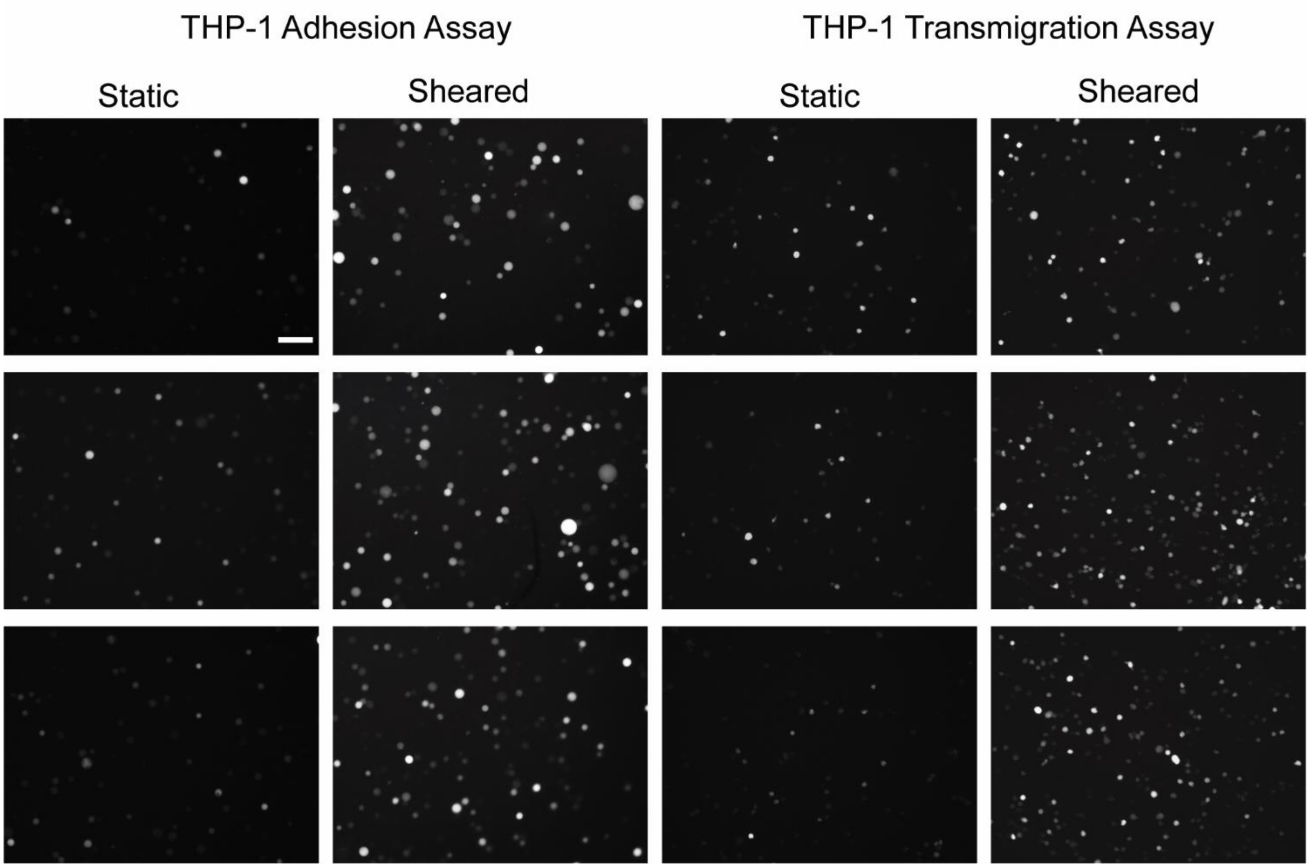

Supplementary Figure 2. Additional images of adhered and transmigrated THP-1 cells were used for quantitative analysis. Scale bar = 100 μm.

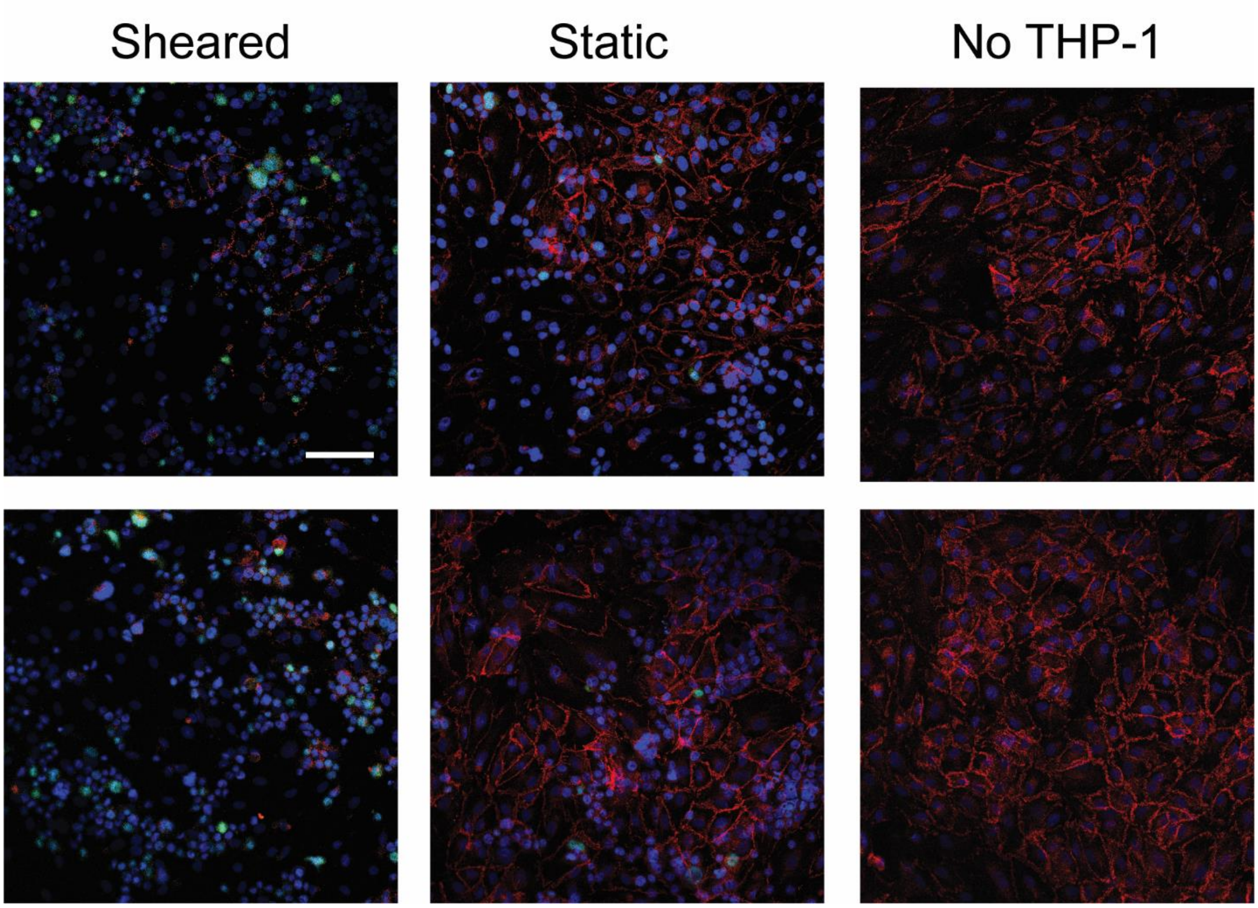

Supplementary Figure 3. Additional images of VE-cadherin staining. Scale bar = 100 μm.

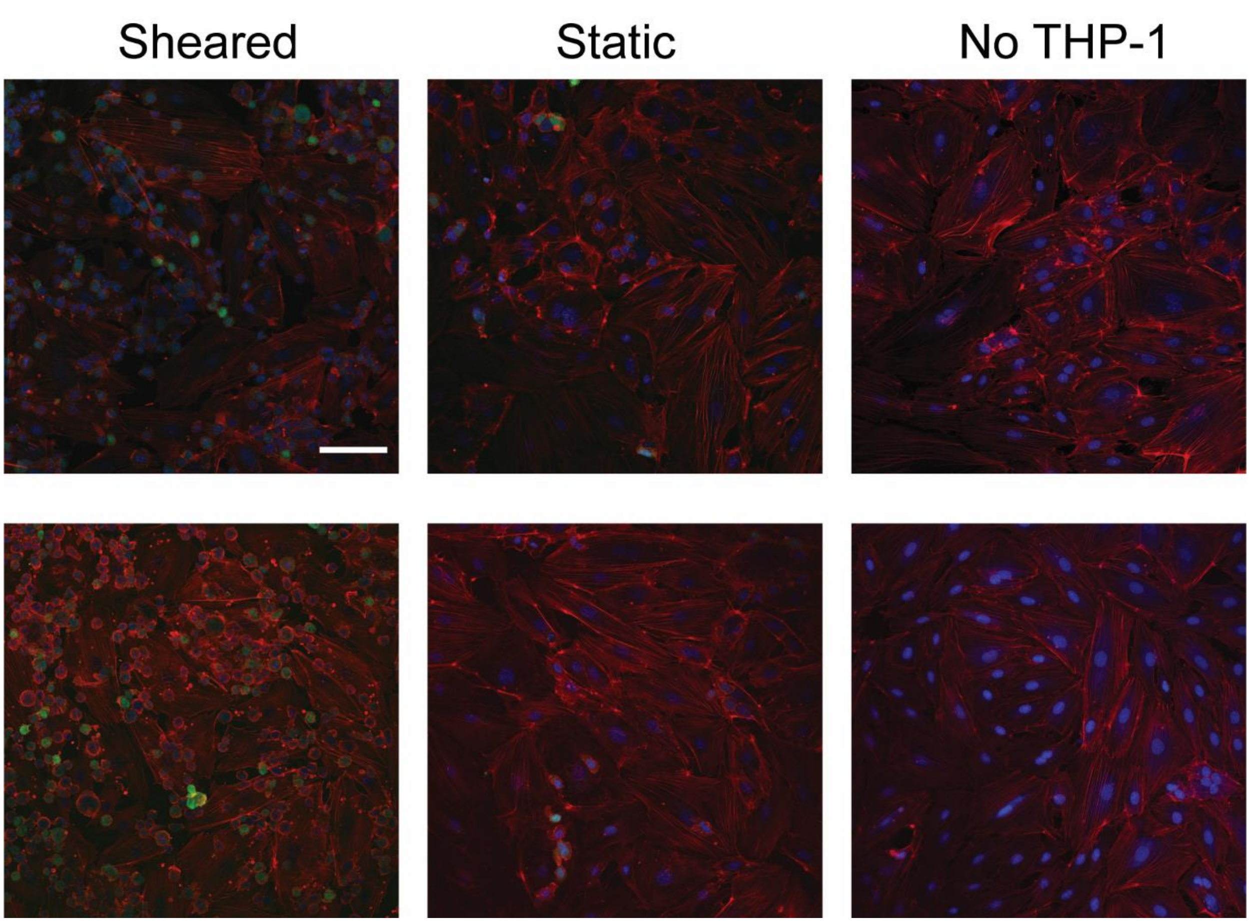

Supplementary Figure 4. Additional images of F-actin staining. Scale bar = 100 µm.

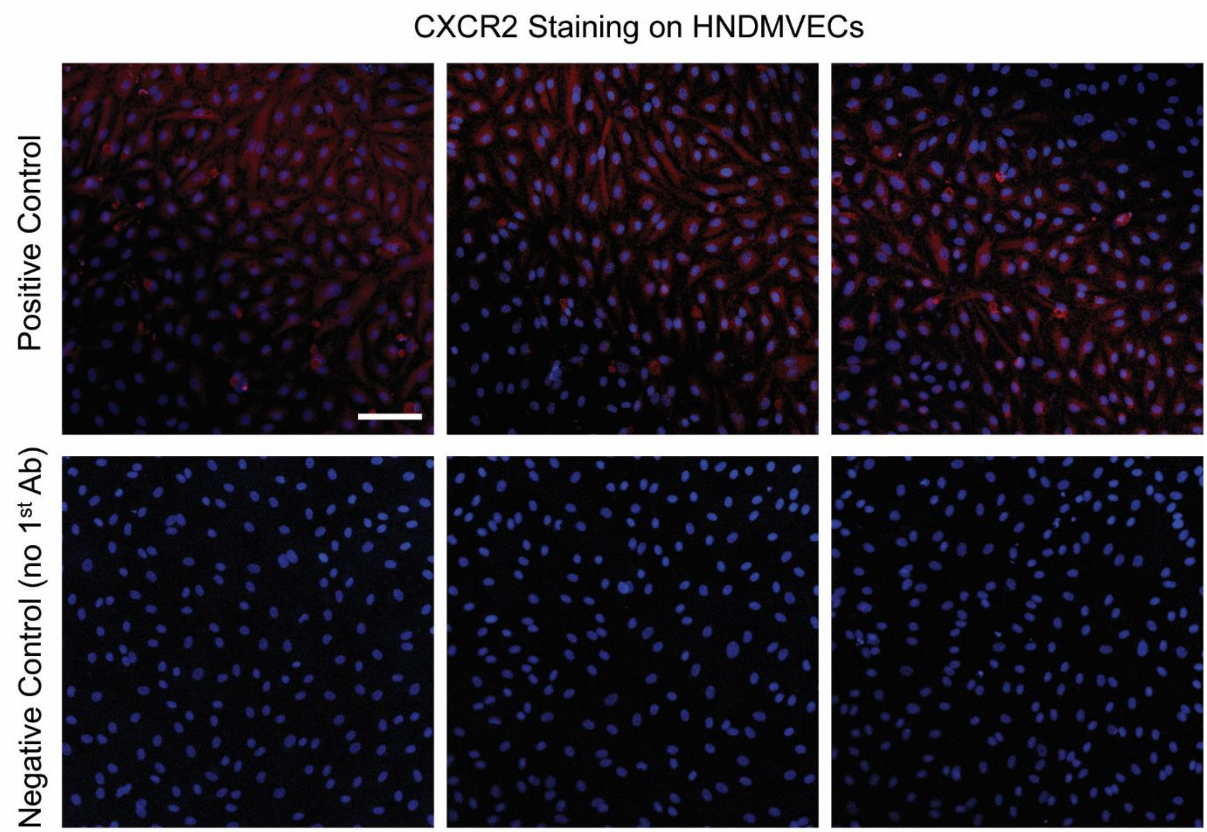

Supplementary Figure 5. CXCR2 staining on HNDMVECs. Scale bar = 100 μm.

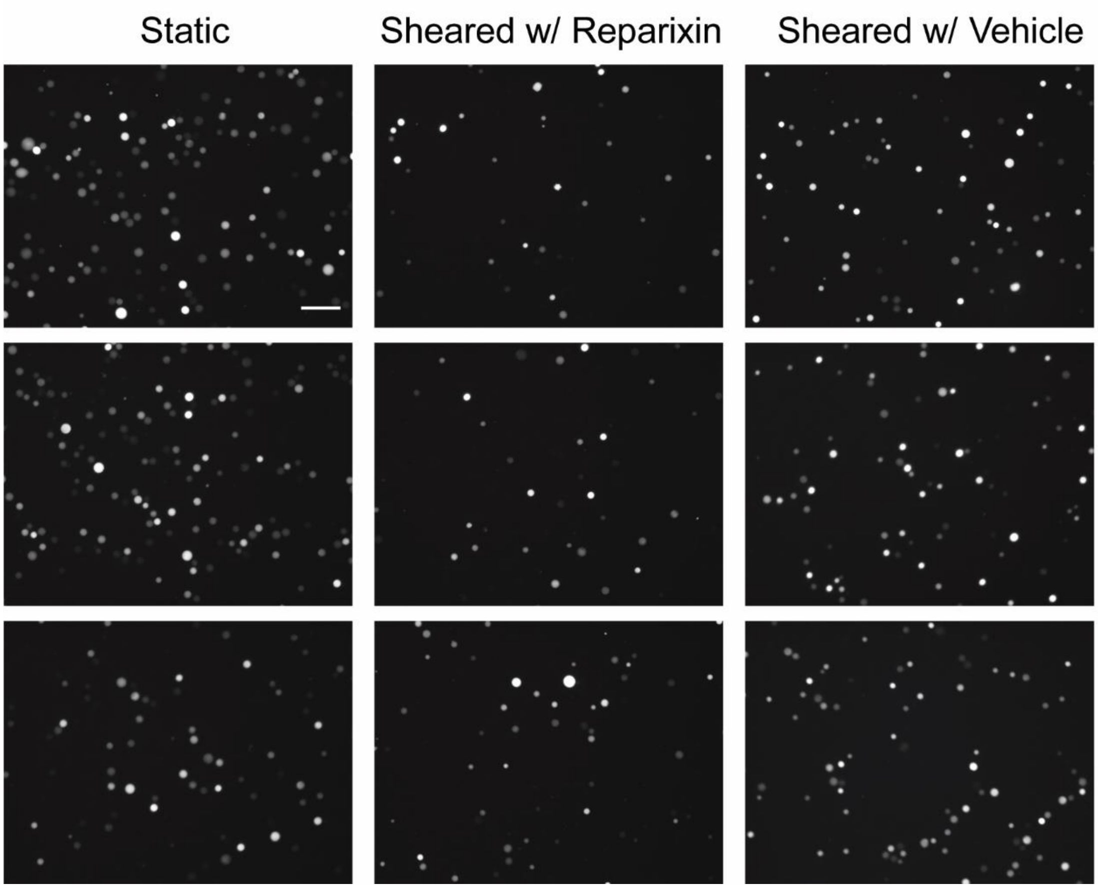

Supplementary Figure 6. Additional images of THP-1 cell adhesion on HNDMVECs treated with or without reparixin. Scale bar = 100 μm.

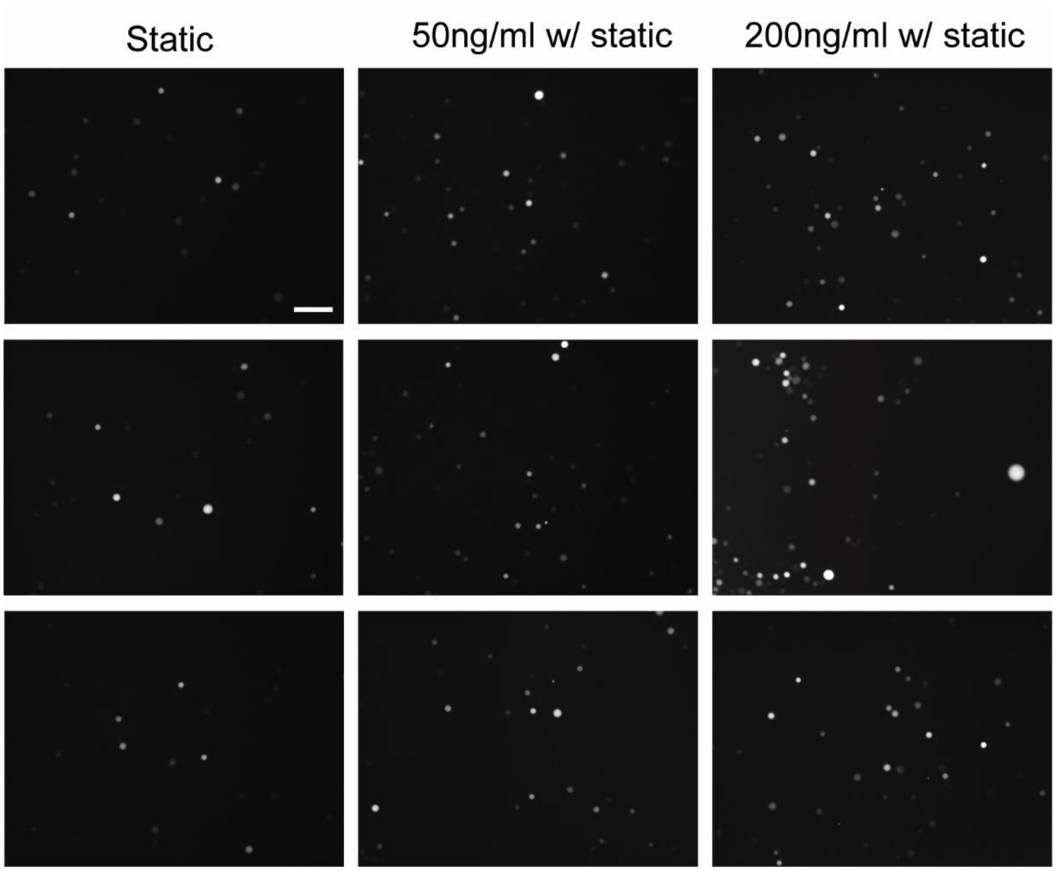

Supplementary Figure 7. Additional images of THP-1 cell adhesion on HNDMVECs treated with or without human recombinant IL-8. Scale bar = 100 μm.

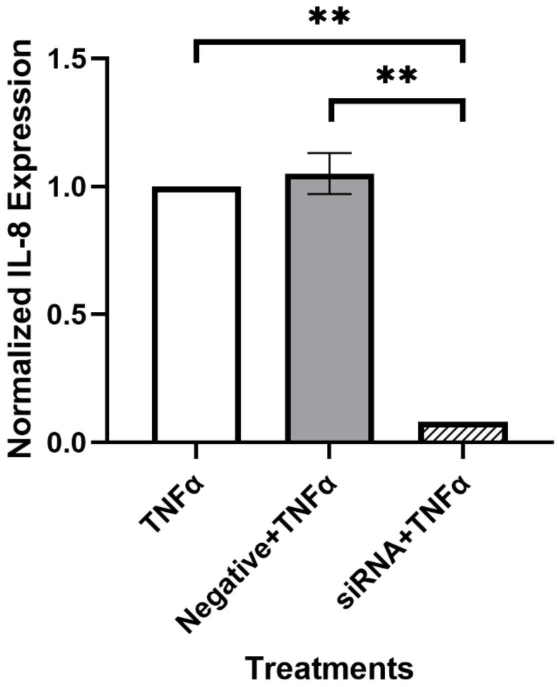

Supplementary Figure 8. IL-8 expression of HNDMVECs treated with TNF-α, negative control siRNA and TNF-α, and IL-8 siRNA and TNF-α.

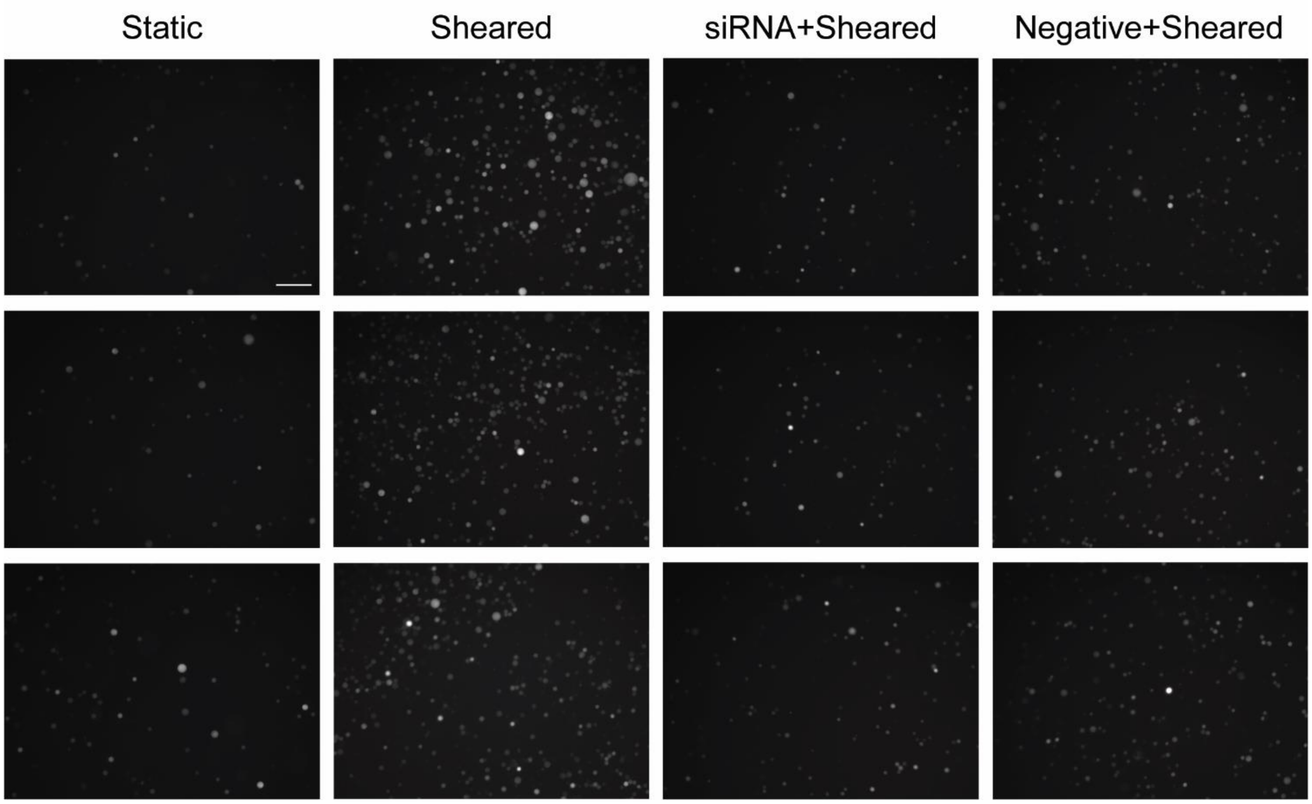

Supplementary Figure 9. Additional images of THP-1 cell adhesion on HNDMVECs treated with siRNA and scramble RNA

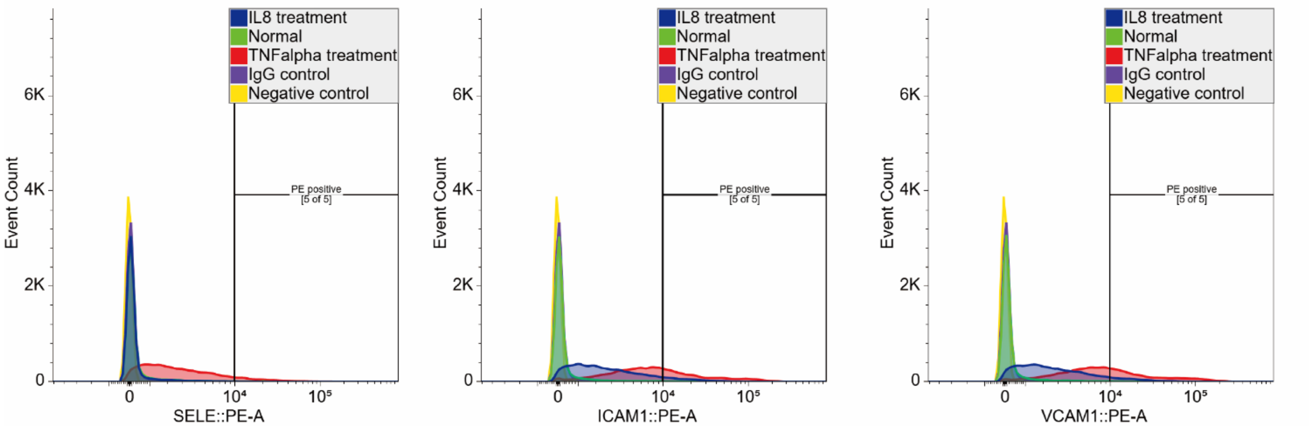

Supplementary Figure 10. FACS analysis for E-Selectin, ICAM, and VCAM adhesion molecules of endothelial cells treated with IL-8 and TNFα as positive control.
